# Ecologic Factors Associated with West Nile Virus Transmission, Northeastern United States

**DOI:** 10.3201/eid1410.071396

**Published:** 2008-10

**Authors:** Heidi E. Brown, James E. Childs, Maria A. Diuk-Wasser, Durland Fish

**Affiliations:** Yale University, New Haven, Connecticut, USA; 1 Current affiliation: University of Oxford, Oxford, UK.

**Keywords:** West Nile virus, spatial epidemiology, GIS, risk factors, research

## Abstract

Since 1999, West Nile virus (WNV) disease has affected the northeastern United States. To describe the spatial epidemiology and identify risk factors for disease incidence, we analyzed 8 years (1999–2006) of county-based human WNV disease surveillance data. Among the 56.6 million residents in 8 northeastern states sharing primary enzootic vectors, we found 977 cases. We controlled for population density and potential bias from surveillance and spatial proximity. Analyses demonstrated significant spatial spreading from 1999 through 2004 (p<0.01, r^2^ = 0.16). A significant trend was apparent among increasingly urban counties; county quartiles with the least (<38%) forest cover had 4.4-fold greater odds (95% confidence interval [CI] 1.4–13.2, p = 0.01) of having above-median disease incidence (>0.75 cases/100,000 residents) than counties with the most (>70%) forest cover. These results quantify urbanization as a risk factor for WNV disease incidence and are consistent with knowledge of vector species in this area.

## Abstract 2

Risk for disease was 4 times greater in the least forested counties.

West Nile virus (WNV) disease arrived in the United States in 1999 in New York City, yet how the disease became established and details concerning the nature of the transmission cycle in the United States remain unclear. Experience in the northeastern United States suggests an urban concentration of human WNV disease cases (*1*,*2*); however, environmental factors, such as urbanization, that underlie the patterns of transmission to humans have not been explicitly evaluated. We used human surveillance data to describe and quantify the spread of WNV cases in the northeastern United States and empirically tested the hypothesis that human WNV disease is linked to the urban environment independent of human population density.

In the northeastern United States, a mainly urban cycle of WNV transmission is supported by the role of bird and mosquito species. This enzootic cycle occurs in urban bird species; human cases occur in late summer (*2*–*7*). *Culex pipiens* Linnaeus is the most commonly implicated mosquito vector in the maintenance of WNV in birds (*1*,*2*,*8*,*9*). In the northeastern United States, this species feeds on birds found in urban areas, such as the American robin (*Turdus migratorius*), house sparrow (*Passer domesticus*), and European starling (*Sturnus vulgaris*) (*2*,*10*). The role of *Cx*. *pipiens* mosquitoes as primary WNV vector is supported by consistent isolations of WNV from mosquitoes captured in surveillance traps (*8*,*11*–*14*) and by associations between virus-infected mosquitoes and dead-bird reports (*15*).

A more contentious issue is the role of different mosquito species in transmitting, or bridging, WNV between birds and other vertebrates, including humans. *Cx. pipiens* mosquitoes are known to breed in the organically rich water of artificial containers frequently found in urban areas (*16*–*18*). Habitat modeling of potential WNV vectors in the northeastern United States indicates an urban focus for *Cx*. *pipiens* mosquitoes (*19*). However, its tendencies to mostly feed on birds make it an unlikely bridge vector, although other researchers have suggested that this species exhibits late season host switching to humans (*5*). *Aedes vexans* and *Cx*. *salinarius* mosquitoes have been implicated as bridge vectors in this region (*1*–*3*) because of their abundance and more nonspecific feeding patterns (*20*). Although both are present in urban areas, other land uses have been found to be more predictive of their distribution (*19*). These other studies do not indicate whether human incidence would be linked to the same ecologic factors driving enzootic transmission.

In this study, we explicitly tested whether both enzootic and bridge transmission occur in urban areas by evaluating human WNV disease and degree of urbanization within counties. We estimated the initial spatial spread in time to first case in Queens, New York, the site of first WNV detection (*21*), from 1999 through 2006. We also examined the trend for increasing incidence with decreasing forest cover while attempting to control for surveillance efforts and removing the effect of spatial proximity. The methods provide an example of how surveillance data with low spatial resolution can be used to quantify risk.

## Methods

The study was focused in 8 northeastern states (Connecticut, Delaware, Massachusetts, Maryland, New Jersey, New York, Pennsylvania, and Rhode Island) where the same mosquito species are likely to act as primary vectors. States to the north of the study area have had limited numbers of cases and may involve different mosquito species. States farther south and west are likely to involve different species of mosquitoes; hybridization between *Cx*. *pipiens* and *Cx*. *quinquefaciatus* is more common in southern latitudes (*16*).

### Human Incidence Data

We used annual numbers of human WNV cases reported to the Centers for Disease Control and Prevention (CDC) from 1999 through 2006. Human case data were acquired through multiple sources but met the CDC case definition, which includes clinical disease with laboratory confirmation. Data for 1999 were extracted from the Morbidity and Mortality Weekly Report (*22*), and data for 2000 were downloaded from the National Atlas website (http://nationalatlas.gov; *23*). Human case data for 2001 through 2006 were downloaded from the US Geological Survey maps page (http://nationalatlas.gov/printable/wnv.html; *24*). To protect anonymity, human data from these sources are compiled at the county level. All other data were aggregated by county to match this resolution.

### Geographic Data

County boundaries for the United States and 2000 census data were downloaded from the National Atlas website (http://nationalatlas.gov/boundaries and http://nationalatlas.gov/people), and county centroids were identified to facilitate the calculation of distances between counties. Land-use data were downloaded by state from the US Geological Survey National Land Cover Institute (http://landcover.usgs.gov/natllandcover.php; *24*). Percentage of land cover class by county was extracted by using Fragstats Software (*25*). Land uses classified as low-intensity residential, high-intensity residential, commercial/industrial/transportation, and urban/recreational grasses were grouped into a class called *urban.* Land uses classified as deciduous, evergreen, and mixed forest were grouped into a class called *forest.* These 2 land use types were considered biologically relevant to the study question.

### Statistical Analyses

To document evidence for the temporal and spatial spread of WNV disease, we generated cumulative incidence curves by state and by year and examined the distance between counties with cases. Time-to-first-case detection (in years) was used as the outcome predicted by distance to the origin, which was Queens, New York. For distance calculations, we ignored counties reporting no WNV disease cases because the first case is theoretically still to be determined. To visualize WNV disease spread, we plotted the mean incidence by year, using the spatial statistics tools of ArcGIS (*26*).

Distance measures were then used to adjust for the effect of spatial proximity in the regression analyses (*27*). Incorporating measures of spatial proximity in a regression model removes the effect of spatial structure that might otherwise result in overestimation of the strength of the association between the outcome, WNV incidence, and the explanatory environmental variables (*28*,*29*).

Logistic regression modeling was initially used to identify the relevant predictors and to quantify their relative effects by calculation of odds ratios (ORs). Number of cases per county was standardized by using the 1990 US Census population density. Cumulative WNV disease incidence data from 1999 through 2006 were dichotomized at their median to provide 2 categories of high and low risk. Predictor variables, percent urban, percent forested, county area, and per capita county income were stratified by quartiles. Logistic models were tested by using the Hosmer-Lemeshow goodness-of-fit test. The best model was selected based on the Akaike information criterion (AIC), which is a measure of fit that accounts for the number of parameters in the model. Models within 2 AIC units are considered comparable; models within 7 AIC units have less support but are still comparable; and models with differences >10 AIC units are not comparable (*30*). The relationship between increasing cases and decreasing percentage of forested land was tested by using generalized least-square regression in STATA (*31*).

A risk model of total incidence was developed by using log (count +1) transformed incidence as the response variable and the variables identified as important in the logistic regression analyses as predictors. To obtain a better fit, predictor variables were entered as continuous values for this regression. The κ statistic was used to assess agreement greater than chance between the median dichotomized original incidence and the predicted incidence, for which <0.21 is considered slight to poor and >0.61 is considered substantial to almost perfect (*32*).

All models were initially run using only the land-use predictors; and the Moran *I* test was used to assess whether closer observations were more similar than those farther apart. This finding of an association based on spatial location could indicate that proximity, rather than environmental factors, explains the distribution of disease incidence. Distance variables control for this potential spatial proximity effect and reflect the presumed biological relationships within the data.

The models were also adjusted for surveillance effort. Human disease surveillance data must be interpreted with knowledge of the biases inherent to its collection (*33*). County per capita income was used as a measure of potential investment in surveillance and laboratory testing, as has been used in prior studies of surveillance for animal rabies (*34*).

## Results

### The Epidemic

**Figure 1:**
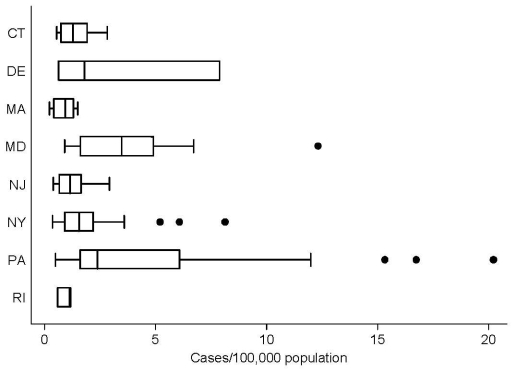
Box plot of total incidence of West Nile virus disease in humans, by county, for the 8 northeastern states in the study area (CT, Connecticut; DE, Delaware; MA, Massachusetts; MD, Maryland; NJ, New Jersey; NY, New York; PA, Pennsylvania; RI, Rhode Island). The box plot provides the median, lower, and upper quartiles; the standard deviation; and any data outliers. This plot excludes those counties that did not report cases. The outliers tend to be the few cases that occurred in areas with low populations.

From 1999 through 2006, the 204 counties in the 8 states reported 977 WNV disease cases (county mean 4.8, SD 8.7, median 1, range 0–49) (Table 1). The median county incidence over the 8-year interval was 0.75 cases/100,000 residents (mean 1.77, SD 3.0, range 0–20.2/100,000). The median incidence, excluding counties with no reported cases, was 1.70/100,000 residents (mean 2.94, SD 3.45, range 0.22–20.2/100,000) (Figure 1). The highest incidence occurred in Forest County, (20.2/100,000), followed by Cameron County (16.8/100,000) and Adams County (15.3/100,000), all rural counties in central Pennsylvania with very few cases (Forest County n = 1, Cameron County n = 1, and Adams County n = 14 [13 in 2003, 1 in 2004]), and small populations, probably representing data outliers.

**Table 1:** Incidence (per 100,000 persons) of West Nile virus disease in humans, northeastern United States, 1999–2006*

State	1999	2000	2001	2002	2003	2004	2005	2006	Mean	Median	25% IQR	75% IQR
CT	0	0.11	0.70	1.97	5.15	0.11	0.7	1.06				
DE	0	0	0	0.79	8.55	0	0.99	0	3.44	1.80	0.64	7.90
MA	0	0	0.44	2.57	2.19	0	0.61	0.27	0.43	0.11	0	0.93
MD	0	0	0.8	9.52	32.01	11.88	1.32	1.69	2.38	1.47	0	3.90
NJ	0	1.02	2.04	7.31	10.04	0.2	0.85	0.68	1.05	0.99	0.43	1.56
NY	3.18	2.45	1.19	21.03	18.78	2.44	2.95	2.03	0.87	0	0	1.25
PA	0	0	0.81	15.87	163.75	7.23	8.36	3.63	2.98	1.59	0	3.09
RI	0	0	0	0.16	2.57	0	0.16	0	0.58	0.60	0	1.13
Total	3.18	3.58	6.01	59.22	243.04	21.76	15.93	9.37	1.77	0.75	0	2.06

*IQR, interquartile range; CT, Connecticut; DE, Delaware; MA, Massachusetts; MD, Maryland; NJ, New Jersey; NY, New York; PA, Pennsylvania; RI, Rhode Island.

### Associations Based on Spatial Proximity

**Figure 2:**
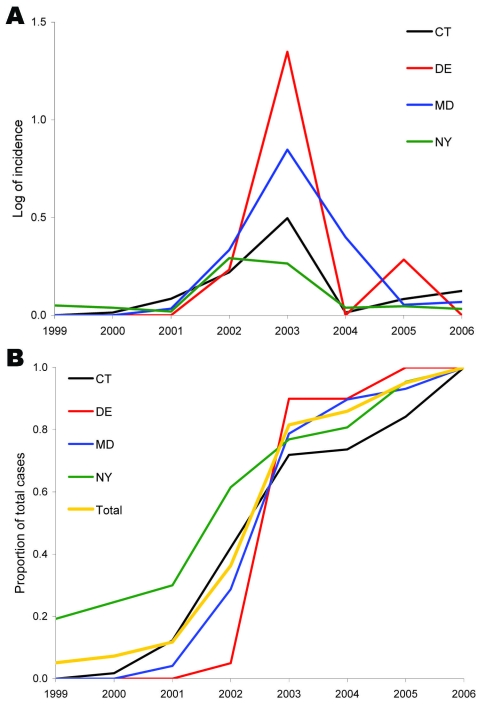
A) Epidemic curve of mean incidence (log+1 transformed) of West Nile virus disease in humans, by state, 1999–2006. The 4 states depicted are representative of the variation among the 8 states in the study area. CT, Connecticut; DE, Delaware; MD, Maryland; NY, New York. This graph shows the trend toward increasing incidence and a regional peak in 2003. NY seems to show a 2-year plateau with similar values for 2002 and 2003. B) Cumulative proportion of total cases for the 8 years also highlighting the 2003 regional peak but suggesting a spatial spread where cases started to rise earlier in NY than in states such as DE that were more distant from the epicenter.

**Figure 3:**
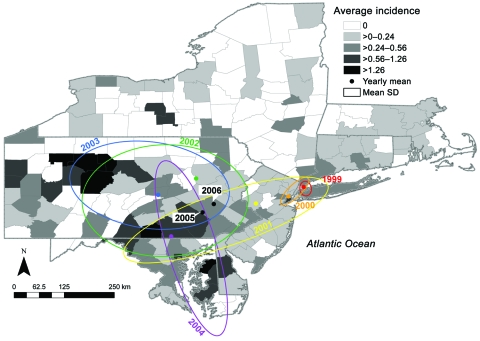
Incidence of human West Nile virus disease cases in 8 northeastern states, 1999–2006. Deviation ellipses indicate 1 SD of the geographic mean yearly incidence calculated as the incidence weighted average in space for each county. Incidence is attributed to the county centroid. This graph shows the urban concentration along the Eastern Seaboard as well as the outliers in western Pennsylvania (1 case in counties with low populations). The 2005 and 2006 regression of the geographic mean incidence is also depicted.

A cursory examination of the epidemic curve of WNV cases reported from each state during the 8-year study indicated that peak incidence was broadly overlapping in all states (Figure 2, panel A). However, cumulative distribution functions of total WNV cases (Figure 2, panel B) by year indicated that New York experienced its median case earlier in the regional epidemic than did other states (Massachusetts, New Jersey, and Connecticut), which suggests a spatiotemporal spread of WNV. Because a spatial component to spread was evident, we evaluated distance between counties to assess the spatial relationship between counties and to control for the effect of spatial proximity. The spatial component alone explained 15% of the variance in time to first case when Queens, New York, was used as the origin (n = 123 counties with cases reported, p = 0.001). After 2004, no new counties reported WNV cases, and the incidence centroids of cases in 2005 and 2006 were close to one another and had shifted back toward the origin, which suggests that the disease may have reached endemicity in the region (Figure 3).

### Environmental Risk Factors

Risk (high or low) for WNV cases was significantly associated (by county quartile) with measures of urbanization and with percentage of forested or urban land. Because these 2 measures were highly correlated, we used only a single measure in the final analysis (Table 2). Total county area and other demographic indices (age) were not significant predictors and are not shown.

**Table 2:** Odds ratios for median split incidence of West Nile virus diseases in humans, for significant variables*

Predictor	Adjusted			Unadjusted	
	OR (95% CI)	Significance		OR (95% CI)	Significance
% Forest land use, per quartile					
1st (<38.29)	4.40 (1.91–10.11)	0.000		4.36 (1.44–13.25)	0.009
2nd (38.29–56.56)	3.09 (1.38–6.92)	0.006		2.86 (1.01–8.06)	0.047
3rd (56.56–69.59)	0.84 (0.37–1.91)	0.675		0.81 (0.33–2.00)	0.644
4th (>69.59)	1	NA		1	NA
% Urban land use, per quartile					
1st (<1.68)	1	NA		1	NA
2nd (1.68–4.66)	1.52 (0.68 - 3.39)	0.309		1.42 (0.54–3.76)	0.478
3rd (4.66–15.13)	2.44 (1.09 - 5.43)	0.030		3.08 (0.94–10.12)	0.064
4th (>15.13)	4.38 (1.91- 10.03)	0.000		7.02 (1.78–27.71)	0.031

*Variables categorized by percent of county classified as forested and percent of county classified as urban. Outcome categorized by median split to counties with low risk (incidence <0.75 cases/100,000 residents) and high risk (incidence >0.75 cases/100,000 residents). Overall trend is for increasing incidence with increasing measures of urbanization (for decreasing percentage forested land: χ^2^ = 9.47, df = 1, p< 0.01, goodness of fit χ^2^ = 3.50, df = 2,  p = 0.17; for increasing percentage urban land: χ^2^ = 7.13, df = 1, p< 0.01, goodness of fit χ^2^ = 1.98, df = 2, p = 0.37). Both unadjusted and surveillance bias and spatial relationship adjusted ORs are provided. OR, odds ratio; CI, confidence interval; NA, not applicable.

A logistic regression of the median split for total incidence with categorical predictor variables of percentage forested area and county-based per capita income showed that percentage of forested land (χ^2^ = 26.13, df = 6, p<0.001) and percentage of urban land (χ^2^ = 5.62, df = 6, p = 0.02) were both significant predictors of incidence (Table 2). Both models provided a good fit (forested: Pearson χ^2^ = 7.82, df = 9, p = 0.55; urban: Pearson χ^2^ = 3.26, df = 8, p = 0.92). No effect of spatial proximity was found among the residuals for either model (forested: Moran *I* = –0.008, Z = –0.49, p = 0.31; urban: Moran *I* = –0.002, Z = 0.40, p = 0.34).

To adjust for surveillance bias and the spatial relationship among proximal counties, we included the variables of county-based per capita income and distance from Queens, New York, respectively (Table 2). Both forested (χ^2^ = 36.67, df = 11, p<0.001) and urban (χ^2^ = 33.55, df = 11, p<0.001) predictors were significantly associated with WNV incidence and provided a good fit (forested: Pearson χ^2^ = 209.27, df = 192, p = 0.19; urban: Pearson χ^2^ = 202.78, df = 192, p = 0.28). As before, no effect of spatial proximity was found in the residuals (forested: Moran I = –0.007, Z = –0.38, p = 0.35; urban: Moran *I* = 0.001, Z = 0.93, p = 0.18). Although all models were significant and fit the data, the latter model was preferred on the basis of AIC (not controlling for spatial proximity AIC_forested_ = 270.7, AIC_urban_ = 281.2; controlling for spatial proximity AIC_forested_ = 264.1, AIC_urban_ = 267.3) and included biologically relevant controls for the effect that spatial proximity might have in estimating the association between the outcome, disease incidence, and environmental variables of interest. A general, dose-dependent trend indicated increasing incidence as measures of urbanization increased (higher incidence with decreasing percentage of pixels classified as forest in each county: χ ^2^ = 9.47, df = 1, p<0.01; goodness of fit χ ^2^ = 3.50, df = 2, p = 0.17; higher incidence with increasing percentage urban land: χ ^2^ = 7.13, df = 1, p<0.01; goodness of fit χ ^2^ = 1.98, df = 2, p = 0.37).

The logistic regression model of dichotomized total incidence for the 8 years of the study, controlling for income (categorical variable by quartile) and for the effect of spatial proximity (distance variables), also showed a distinct trend of increasing incidence with percentage of forest cover; counties with <38% forest cover were 4.4× more likely (95% confidence interval 1.4–13.2, p = 0.01) to have high WNV incidence than were counties with >70% forest cover (Table 2).

### Predictive Model

We used the predictors identified in the logistic regression analysis to develop a linear regression model to predict total incidence (log count + 1 transformed for a normal distribution), using the quartile percent forested land by county. Per capita income (as a continuous variable) was used to control for surveillance effort. This model explains 9.7% of the variance in the total incidence (log count + 1) (p<0.001); however, the residuals indicated an effect due to spatial proximity (Moran *I* = 0.0349, Z = 5.925, p<0.001). Controlling for this spatial effect and surveillance effort resulted in a better model (*r*^2^ = 0.20, p<0.001; Moran *I* = –0.003, Z = 0.26, p = 0.40). The κ statistic indicated good agreement (κ = 0.343, SE = 0.066, Z = 5.22, p<0.001, agreement = 67.16%) between the predicted and the observed outcomes when the binomial categorization of incidence was used and resulted in 51 county incidence entries being correctly identified as being below the median and 86 being correctly identified as being above the median. Errors were primarily in the direction of predicting the incidence above the median. When surveillance and spatial proximity were controlled for, the risk for WNV disease increased by 0.25% for every 1% decrease in forest cover. For more direct comparison with the logistic regression outcome, moving from the highest category of forest cover (>69.59%) to the lowest (<38.29%), resulted in a 6.16% increased risk for WNV disease.

## Discussion

This study documents the concentration of WNV cases within urban areas of the northeastern United States and provides a quantitative estimate of the effect of varying degrees of urbanization on the risk for WNV infection at the county level. Land-use data were used to ascribe degree of urbanization as a predictor for WNV disease risk; incidence models were generated, controlling for human population density, environment-based spatial associations in the predictors, and potential biases in WNV incidence reporting resulting from the unequal resource bases among counties.

Beginning in 1999, human WNV cases were reported in counties distant from Queens, New York, the presumed origin of infection. Although the epidemic initially appeared to spread in a west/southwesterly direction in the 8-state region examined, by 2005 the initial epidemic appeared to wane, and reports of disease among newly affected counties dropped to zero. The resulting incidence maps suggest a WNV disease–endemic situation in the northeastern United States. The initial spread was not continuous along neighboring counties; rather, greater incidence was seen in urban counties after controlling for human population density, surveillance bias, and the effect of spatial proximity. The best model indicates 4× the risk for disease in the counties that fall in the lowest incidence quartile of forested land compared with the highest. The predictive nature of the data is also explored with the caveat that additional predictor variables are needed; nonetheless, it indicates increasing risk for WNV disease with decreasing forested lands.

The association between urban land use and human cases indicates that urban/suburban land use enhances environmental conditions for both enzootic and bridge transmission, at least at the county level. The spatial resolution of human surveillance data did not allow for finer evaluation of within-urban associations. Brownstein et al. linked human WNV cases to greenness indices in urban areas and found an optimal vegetation index associated with higher human cases (*35*). Brown et al. found an environmental separation of bridge and enzootic vectors; bridge vectors occurred in areas with vegetation that might be associated with residential areas within a city (*36*). Finer spatial resolution human data would allow for within-county analyses that might provide better estimations of where the cases (urban, periurban) are occurring. This would improve the predictive power of land use in the models, and the better association between land use and cases might help further elucidate which mosquito species are involved as bridge vectors.

Because of the type and resolution of the data, a sample predictive model, and not a predictive map, is provided. Nonetheless, the data and analysis provided are insightful as potentially predictive models. Additional data, such as bird abundance and perhaps also mammal abundance, are needed (*37*). Because of the often strict host and habitat preferences of mosquito species, mosquito surveillance data could also improve the predictive power and validity of the model. Our best predictive model explains only 20% of the variance; additional variables such as these might improve the model because the abundance of hosts and mosquito species will have a considerable effect on WNV transmission.

Despite the reluctance to use human surveillance data for models of disease transmission (*33*), such data can provide information about spatial associations in vector-borne disease as shown here and by others (*34*,*38*,*39*). This type of human surveillance modeling provides some useful insight into the distribution of human WNV cases and supports the current understanding of the transmission cycle.

To predict WNV disease requires understanding of the factors driving both enzootic transmission and bridging to humans. Different data availability and scales are involved in studying these 2 processes. We took advantage of the national coverage of the human incidence dataset to examine the spatiotemporal spread of WNV in this region and to generate a risk model based on land use, adjusted for the effect from spatial proximity. We show that human surveillance data at the county level are consistent with the urban nature of this disease system, as has been found in studies of enzootic transmission, indicating that the 2 processes occur in or near urban areas.
